# Feasibility and effectiveness of interpersonal psychotherapy interventions in a collaborative stepped care model between primary care and mental health services

**DOI:** 10.1192/j.eurpsy.2021.1331

**Published:** 2021-08-13

**Authors:** F. Mongelli, F. Martino, D. Berardi, N. Colombini, S. Ferrari, M. Menchetti

**Affiliations:** 1 Department Of Medical And Surgical Sciences, University of Bologna, Bologna, Italy; 2 Department Of Mental Health, Community Mental Health Centre Ravenna, Ravenna, Italy; 3 Department Of Mental Health, Modena, Local Health Unit Castelfranco Emilia, Castelfranco Emilia, Italy; 4 Department Of Biomedical, Metabolic And Neural Sciences, University of Modena and Reggio Emilia, Modena, Italy

**Keywords:** Interpersonal Psychotherapy, Mental Health Services, collaborative stepped care model, primary care

## Abstract

**Introduction:**

The NICE guidelines recommend for mild major depression a range of low-intensity psychosocial intervention of proven effectiveness, as Interpersonal Counselling, and a stepped-care approach.

**Objectives:**

To assess feasibility and effectiveness of Interpersonal Psychotherapy interventions for the treatment of depression in a consolidated Collaborative Stepped Care Model between primary care and mental health specialists.

**Methods:**

103 patients were referred by their PCPs to the Consultation-Liaison Service of Bologna and Modena. Of them, 78 were included in the study and administered self-report instruments and interview, including screening depression, anxiety and daily functioning. Patients were asked to choose one of the available treatment: 1) Interpersonal Counseling (IPC) 6-8 weekly meetings for 30 minutes; 2) IPC for Depression in Primary Care 3 sessions of 50 minutes; 3) a guided self-help intervention. Follow-up were planned at 1, 3 and 6 months. Both patients and PCPs provided a feedback about intervention’s satisfactions.

**Results:**

At the baseline, 39.4% of the patients presented a minor depression/major depression mild and the large majority (75.0%) of them chose IPC, while none of them chose the guided self-help intervention. At follow ups the mean PHQ-9 significantly decreased compared to the baseline (p<0.001); daily functioning increased (WSAS: p<0.001) and anxiety traits improved (STAI: p<0.001). Patient’s general satisfaction with the service received was high (GSQ: 85.9±15.0) as well as PCPs, 62.7% of them expressed high satisfaction for the intervention.

**Conclusions:**

The study emphasised that IPC is an effective and feasible treatment very well suited to the primary care setting for an optimal management of depression.

